# Assessment of image rejection in digital radiography

**DOI:** 10.25122/jml-2022-0341

**Published:** 2023-05

**Authors:** Mohamed Hasaneen, Noora AlHameli, Amel AlMinhali, Shamma Alshehhi, Suliman Salih, Mohammad Mubarak Alomaim

**Affiliations:** 1. Department of Radiology and Medical Imaging, Fatima College of Health Sciences, Abu Dhabi, United Arab Emirates; 2. King Saud bin Abdulaziz University for Health Sciences, Riyadh, Saudi Arabia

**Keywords:** reject analysis, medical imaging, ionizing radiation

## Abstract

X-ray imaging uses ionizing radiation to generate diagnostic images. However, unnecessary radiation exposure can pose potential risks, including an increased risk of malignancy. One factor contributing to unnecessary radiation exposure is the rejection and retaking of X-ray images, which can lead to higher patient and occupational radiation doses. This study aimed to assess digital radiography rejection rates, causes of recurrence, and the most commonly repeated types of examinations. A cross-sectional online-based survey was conducted in 2022, involving 62 randomly selected radiographers in the UAE. The survey was distributed to radiographers through the head of radiology departments in various hospitals. Hospitals agreed to participate in the survey without disclosing their name. The data collected was analyzed using Excel. The study showed that 71% of radiographers working in the UAE hold a bachelor's degree. The examinations most frequently repeated were related to anatomical areas, with the spine accounting for 37.7% and facial bone for 19.7% of cases. The factors influencing repetition were primarily related to positioning (48.4%) and artifacts (21%), with the motion being the main cause of artifacts, including voluntary and involuntary movements. This study concluded that the most prevalent cause of repeating and retaking images is positioning, followed by artifacts. Furthermore, night shifts and workload impact radiographer performance, increasing the likelihood of picture retakes. The average number of rejects and repeated images has been reduced as new generations and modern equipment have been introduced, which also helped decrease the numbers.

## INTRODUCTION

Medical imaging plays a critical role in healthcare, encompassing various aspects such as screening, diagnosis, treatment planning, and monitoring [1]. Radiology departments in every hospital uphold to provide images of optimum quality while keeping patients safe from unnecessary ionizing radiation concerning ALARA (as low as reasonably achievable) principles. X-ray, CT (computed tomography), and mammography are commonly used modalities that employ ionizing radiation to produce diagnostic images. This type of radiation can cause biological effects, and overexposure can consequently increase the risks of adverse health effects, including stochastic effects and malignancies [2].

Rejects, deletions, and repeated images in radiological imaging raise concerns regarding professional and ethical standards, highlighting potential shortcomings in quality control [3]. A rejected image is an unacceptable radiograph with inadequate diagnostic value regarding image quality, evaluated by technologists based on technical qualities [4]. Repeatedly taking X-ray images exposes patients to unnecessary ionizing radiation, which poses a substantial risk of cancer development even at prolonged low-dose exposure. However, the overall benefits of medical imaging examinations are believed to outweigh the associated risks, except in cases where redundant examinations are conducted. Therefore, it is crucial to actively reduce the need for image retakes, as it helps minimize X-ray exposure, mitigates patient inconvenience, and optimizes the allocation of medical resources for quality assurance purposes in healthcare settings [5]. The analysis of rejected images serves as a foundation for identifying the reasons for image rejection, and it is also used to evaluate radiographer training and workflow for quality assurance. Moreover, it helps in the safe use of radiation by considering the amount of ionizing radiation a patient is exposed to and the quality of the generated image, so it should be included as part of all quality control and assurance programs [4]. Risk analysis helps identify other underlying issues, such as deficiency in staff training and increasing the department's performance by decreasing retakes to reduce waiting time [2].

With the shift from film-based imaging to digital imaging, it was assumed that the rejection rate (RR) would drop significantly, yet the rate from previous studies is as high. The RR in film-based imaging was between 10% and 15%, the primary cause being incorrect exposure due to limitations in dynamic ranges [3,6]. However, more recent studies on digital imaging by Hofmann *et al*. [3], Rastegar *et al*. [6], Stephenson-Smith *et al*. [4], and Andersen *et al*. [7] showed RRs of 11%, 8%, 9%, and 12%, respectively, with positioning errors being the primary reason for rejection. A research set of lateral knee images was presented to several radiologists and radiographers to study if there were different perspectives on image quality and how it impacts RR. The study showed that images could be prematurely rejected due to diagnostic versus technical evaluation used by radiologists and technicians, correspondingly, which was the main cause of inconsistency of image rejection [2]. Considering the persistently high RR observed in previous reject analyses and the varying reasoning among radiologists and radiographers, quality management becomes crucial in maintaining control and ensuring quality assurance in hospitals.

No previous studies on radiology quality management or reject analysis specific to the United Arab Emirates (UAE) have been identified. A study by Ali and Mohammed [8] in the neighboring country Qatar showed an optimistically very low rate of 0.78%, with patient movement as the main cause. However, the sampling size of the study was very small compared to studies done in other countries. Another study conducted in Saudi Arabia [9] aimed to analyze the RR and found a rate of 8.96%, with the pelvis being the anatomical area associated with the highest rejection rate.

The purpose of this study was to assess digital radiography rejection rates and causes of recurrence and determine the most commonly repeated types of tests.

## MATERIAL AND METHODS

A quantitative study was conducted in March 2022, focusing on radiographers working in hospitals across the UAE. The sample consisted of 62 radiographers randomly selected to participate in the study. There were no specific criteria regarding age or years of work experience for inclusion in the study. The main instrument used to collect primary data for this study was an online cross-sectional survey of 20 closed questions divided into three sections (see Appendix 1). The first section of the survey was the demographic section which contained five closed questions focusing on background information such as educational level and work experience. The second section (reject and repeat) focused on identifying the reasons for a radiographer to repeat or reject an image. Lastly, the quality assurance section aimed to find and understand the role of management in controlling and managing cases of increased RRs. The survey was distributed to the heads of radiology departments in different hospitals across the UAE, who then forwarded it to their radiographers. Participating hospitals agreed to take part in the survey while maintaining confidentiality.

Quantitative data analysis was performed using Excel software. Descriptive and comparative analyses were conducted to assess similarities and differences in rejection rates and interpretations.

## RESULTS

### Sample size

The study collected a total of 62 survey responses from radiographers working in both public and private hospitals across the UAE in March 2022.

### Repeated images by reasons and factors

Table 1 presents the findings on the reasons for repeating examinations, as identified in this study. The most common reasons for repeating examinations were improper positioning (48.4%), the presence of artifacts (21%), and wrong exposure factors (9.7%), while the least common reasons were collimation (1.6%) and markers (3.2%). Additionally, radiographers agreed that workload (79%) and night shifts (51.6%) directly affect the number of repeated images and their performance, as shown in Table 2.

**Table 1.** Reasons for repeating the examination

Reasons for repeating the examination	Responses (%)
Positioning	48.4%
Exposure	9.7%
Gridline	8.1%
Artifacts	21%
Collimation	1.6%
Marker	3.2%
Central ray	8.1%

**Table 2.** Workload and night shifts as reasons for repeating examinations

Question	Yes	No	Not applicable
Does workload have a direct effect on the number of repeated images?	79%	21%	0
Do night shifts affect your performance?	51.6%	46.8%	1.6%

As artifacts were the second main factor in repeating the procedure, Table 3 presents the distribution of reasons for various artifact types that occur in images. Motion artifacts (42.6%) were the most common, while radiopaque objects (13.1%) were the second main type of artifact. Grid cut-off artifacts, backscatter, and overexposure/underexposure artifacts each accounted for 11.5%. Image compositing artifacts (3.3%) were the least common type. Moreover, 6.6% of radiographers chose other artifacts.

**Table 3.** Common artifacts for repeating examinations

Common artifacts	Responses (%)
Motion artifact	42.6%
Radiopaque objects	13.1%
Grid cut-off artifact	11.5%
Backscatter	11.5%
Over/underexposure	11.5%
Image composition	3.3%
Others	6.60%

### Repeated images by anatomical area

The RRs by body area are shown in Table 4. The number of repeated images per anatomical area is shown in Table 5. Spine examinations (37.7%) accounted for the majority of repeats, according to the radiographers, followed by facial bone (19.7%), pelvis/abdomen (16.4%), thorax/chest (14.8%), and extremities (11.5%). As Table 5 demonstrates, the most challenging areas to examine, according to the radiographers, are facial bone (29.5%), pelvis/abdomen (24.6%), and spine (21.3%), while extremities (16.4%) and thorax/chest (8.2%) are the least challenging. This was expected according to the most frequently repeated examinations by body area.

**Table 4.** Repeated images by anatomical area

The body part most repeated	Responses (%)
Extremities	11.5%
Pelvis/abdomen	16.4%
Spine	37.7%
Thorax/chest	14.8%
Facial bone	19.7%

**Table 5.** Most challenging anatomical areas

Most challenging anatomical areas	Responses (%)
Extremities	16.4%
Pelvis/abdomen	24.6%
Spine	21.3%
Thorax/chest	8.2%
Facial bone	29.5%

### Role of quality assurance in repeated images

Table 6 highlights that a significant majority of participant radiographers (91.9%) reported adhering to the principle of ALARA, which emphasizes keeping radiation exposure as low as reasonably achievable. In Table 7, it is observed that 69.4% of the radiographers have frequently attended training and refresher courses on radiation protection. Regarding the approach at the department level to monitor repeated and rejected images, 95.2% of radiographers confirmed that their radiology departments perform such checks and maintain documentation. Additionally, 88.7% of the radiographers do routine tests on the machine for quality assurance purposes, and the remaining 11.3% do not.

**Table 6.** Concept of ALARA

Q1	Always	Usually	Sometimes
How often do you follow the concept of ALARA?	91.90%	6.50%	1.60%

**Table 7.** Training and refresher courses on radiation protection

Q1	Yes, frequently	Yes, seldom	No, never
Have you ever attended training and/or refresher courses on radiation protection?	69.40%	25.80%	4.80%

### Role of equipment in repeated images

The role of the equipment used in the rate of repeated images is demonstrated in Table 8. Radiographers were asked three questions on this subject. For the first question, 62.9% of radiographers agreed that digital radiography reduced the percentage of rejected and repeated images, 29% agreed that errors still exist, and 8.1% disagreed. For the second question, 79% absolutely agreed that the generation of the equipment affects the efficiency of the machine, while 21% slightly agreed. Additionally, 95.1% believed that the modern version of the machines performs better than the older ones.

**Table 8.** Role of equipment in repeated images

Q1	Yes	Yes, but errors still exist	Never
Does digital radiography reduce the percentage of rejected and repeated images?	62.90%	29%	8.10%
**Q2**	**Yes, absolutely**	**Yes, not much**	**No**
Does the generation of the equipment affect the efficiency of the machine?	79%	21%	0%
**Q3**	**Yes**	**No**	**-**
From your point of view, does the modern version of the machines perform better than the older ones?	95.10%	4.90%	-

### Role of experience in repeated images

Table 9 shows the relationship between radiographers' experience and the number of repeated images per day. It presents the distribution of repeated images among radiographers with less than 1 year to 5 years of experience compared to those with 6 to 10 years of experience. Among radiographers with less than 1 year to 5 years of experience, 16.6% reported no repeated images per day. The majority of radiographers in this group (73.3%) repeated 1 to 5 images per day. A smaller percentage repeated 6 to 10 images (6.6%), and even fewer repeated 11 to 15 images (3.3%). On the other hand, among radiographers with 6 to 10 years of experience, a slightly higher proportion (21.8%) reported no repeated images per day. The majority of radiographers in this group (78.2%) repeated no more than 1 to 5 images per day.

**Table 9.** Role of experience in repeated images

Number of repeated images	Experience	
	Less than 1 year to 5 years	6 to 10 years
0	16.60%	21.80%
1 to 5	73.30%	78.20%
6 to 10	6.60%	0
11 to 15	3.30%	0

### Number of images taken vs. number of repeated images per shift

The approximate number of images taken per shift by the radiographer is presented in Table 10. The highest number stated is more than 30 (46.8% of radiographers), followed by 21 to 30 images (22.6%), 11 to 20 images (19.4%), and 1 to 10 images (11.3%).

**Table 10.** Number of images taken per shift

Approximate number of images taken per shift	1 to 10	11 to 20	21 to 30	30+
	11.30%	19.40%	22.60%	46.80%

Table 11 presents the average number of images repeated by the radiographers per shift. The most frequent answer, given by 77.4% of the radiographers, was 1 to 5 images, followed by no repeated images (19.4%), and 6 to 10 and 11 to 15 repeated images (1.6% each), while no radiographer stated that the repetition of more than 15 images per shift.

**Table 11.** Number of repeated images per shift

Approximate number of images taken per shift	0	1 to 5	6 to 10	11 to 15	15+
	19.40%	11.30%	19.40%	22.60%	46.80%

## DISCUSSION

This study aimed to examine the incidence of repetitive X-ray examinations among patients and investigate the underlying factors contributing to this issue. Some radiographers rejected images that could be enhanced through post-processing, often resulting from inadequate supervision and inspection. Data were collected in March 2022 from a sample of radiographers working in private and government hospitals across the UAE. Out of the 62 participants surveyed, 53% reported working in private hospitals, while 47% were exclusively employed in government hospitals.

The survey findings revealed common reasons for the repetition of patients' examinations. The primary cause, identified by 48.4% of radiographers, was positioning error, consistent with previous research where positioning accounted for the majority of rejected images (67.1%) [4], highlighting the importance of proper patient instruction to minimize positioning errors. The next most common reason, identified by 21% of respondents, was artifacts. An artifact on an image is a feature that does not correlate with the physical properties of the subject being imaged and may confound or obscure the interpretation of that image [10]. Collimation and markers were the least mentioned causes of image repetition, being mentioned by 3.6% and 1.2% of radiographers, respectively. This suggests that most radiographers were aware of collimating the region of interest and placing markers correctly using digital post-processing.

As the occupational workload increases, it is reasonable to expect a decrease in efficiency. Subsequently, around 79% of radiographers agreed that workload directly affects the number of repeated images. To improve efficiency, it is recommended to provide technologists with regular breaks so they can resume their duties with higher productivity. In some hospitals where the night shift is applicable, around 51% of radiographers agreed that night shifts affect their performance due to the late time and long duty hours. Night shifts should be optional for radiographers, not mandatory.

We identified that the presence of artifacts is one of the major reasons for repeating an examination and exposing patients to additional radiation doses. The primary reason is motion artifacts (42.6%) due to patient movement and lack of instruction. Involuntary movements, such as breathing and cardiac ones, are harder to manage. A previous study highlighted that nearly 20% of the 192 exams assessed were affected by motion artifacts to such an extent that they had to be repeated. For the hospital, this translated to $115,000 in lost revenue for that week in the X-ray department alone [11].

Most radiographers (19.7%) struggle to image facial bone due to the patient's movement and instability and considered it the most challenging body part to examine (29.5%). Patients need to be educated and instructed well before exposure and minimize the occurrence of unwanted radiation. The present study revealed that 37.7% of radiographers had to repeat spine examinations, highlighting the need for focused attention and clear instructions, particularly for post-surgery and trauma patients who often undergo such examinations. A previous study reported that the most rejected examinations in two digital radiography departments were cervical spine and lumbosacral ones, inappropriate positioning being the most common cause of rejection in both cases. Therefore, radiographers at the two centers may need more training in both types of examination [6]. In this study, according to the radiographers’ responses, only 11.5% of them had to repeat examinations of the extremities, indicating that they are relatively easier and less frequently repeated due to the established good practices in this area. The most challenging type of examination, according to the participants, is that of the abdomen/pelvis (24.6%) because of patients’ different sizes and body shapes. However, the least challenging examination is the chest X-ray (8.2%), which indicates that the chest is the easiest body part to examine.

Around 95.2% of government and private hospital radiology departments review repeated and rejected images, documenting the data to improve departmental practices and service quality. Moreover, 91.90% of radiographers follow the concept of ALARA, one of the best concepts to provide radiation protection and reduce radiation dose. Around 88.7% of radiographers do routine tests on the machine for quality assurance and to ensure that the machine is well-prepared to examine the patients. 95.10% of participants agreed that the modern version of the machines performs better than the older ones because it has better updates and the latest technology.

Most radiographers examine more than 30 patients per shift (46.8%), indicating a well-managed workflow in the department. However, this high patient volume can also impact the energy and effort exerted by the radiographers. A significant percentage (77.4%) of radiographers reported repeating 1-5 images daily, suggesting a potential for improvement in this area. To address this, radiographers would benefit from training and refresher courses on radiation protection, as mentioned by 69% of participants who had attended such courses.

This research has several limitations that should be considered. Firstly, the data collection period was relatively short, and the study was conducted exclusively within the UAE. To enhance the validity and generalizability of future studies, it is recommended to extend the data collection period and include a larger sample size encompassing multiple years. Secondly, the presence of department-specific variations in protocols and imaging systems can also impact the reproducibility of the study.

## CONCLUSION

This research has provided valuable insights into the factors contributing to the repetition and retaking of images in radiology departments. The findings highlight the prominent role of positioning errors and artifacts as common causes of image retakes. Moreover, night shifts and workload affect radiographers’ performance, which leads to a high possibility of retaking images. The most common artifacts that led to retaking images were motion artifacts due to the patient's movement. The second most common type of artifact is the presence of radiopaque objects within the images, which can result in misdiagnoses. This study also found that the three most highly challenging anatomical areas, according to the radiographers, the pelvis/abdomen, spine, and facial bone, are those for which examinations are most repeated. The authors highly recommend establishing an RR specifically in the UAE, covering all imaging departments in government and private hospitals and clinics, to improve performance and improve patient dose optimization. Mandatory and concentrated training for quality improvement projects needs to be held.

## Appendix

### I. Demographic questions

1. Gender:
  - Male
  - Female
2. Age:
  - 20–30
  - 31–40
  - 41–50
  - 51–60
3. What is your educational level?
  - Higher diploma
  - Bachelor
  - Master
  - Doctoral
4. How long have you been working in this field?
  - Less than 1 year
  - 1–2 years
  - 3–5 years
  - 5–10 years
  - More than 10 years
5. Do you work in a private or government hospital?
  - Private
  - Government

### II. Reject and repeat

1. Which X-ray examinations do you usually have to repeat?
  - Extremities
  - Pelvis/abdomen
  - Spine
  - Thorax
  - Facial bone
2. Among the following options, please indicate the most common reason you tend to repeat examinations:
  - Positioning
  - Exposure
  - Gridline
  - Artifacts
  - Collimation
  - Marker
  - Central ray
  - Other, specify ______
3. In your experience, what is the most challenging procedure in general radiography?
  - Extremities
  - Pelvis/abdomen
  - Spine
  - Thorax
  - Facial bone
4. What is the most common artifact seen in the images?
  - Motion artifact
  - Grid cut-off artifact
  - Image compositing
  - Radiopaque object
  - Backscatter
  - Overexposure/underexposure
  - Other, specify ______
5. Does workload have a direct effect on the number of repeated
  - images?
  - Yes
  - No
6. Do night shifts affect your performance?
  - Yes
  - No
7. On average, what is the approximate number of patients you examine per shift?
  - 1–10
  - 11–20
  - 21–30
  - More than 30
8. Approximately how many examinations do you repeat in a day?
  - 0
  - 1–5
  - 6–10
  - 11–15
  - More than 15

### III. Quality assurance

1. How often do you follow the concept of ALARA?
  - Always
  - Usually
  - Sometimes
2. Have you ever attended training and/or refresher courses on
  - radiation protection?
  - Yes, frequently
  - Yes, seldom
  - No, never
3. Does the radiology department check on the repeated and rejected images and document the data?
  - Yes
  - No
4. Do you do routine tests on the machine for quality assurance
  - purposes?
  - Yes
  - No
5. Has digital radiography reduced the percentage of rejected and repeated images?
  - Yes
  - Yes, but errors still exist
  - Never
6. Does the generation of the equipment affect the efficiency of the machine?
  - Yes, absolutely
  - Yes, not that much
  - No
7. From your point of view, does the modern version of the machine perform better than the older ones?
  - Yes
  - No
